# Searching Synergistic Dose Combinations for Anticancer Drugs

**DOI:** 10.3389/fphar.2018.00535

**Published:** 2018-05-22

**Authors:** Zuojing Yin, Zeliang Deng, Wenyan Zhao, Zhiwei Cao

**Affiliations:** Shanghai Tenth People’s Hospital, School of Life Sciences and Technology, Tongji University, Shanghai, China

**Keywords:** synergistic combination, optimized dose combination, computational model, feedback system control scheme, regression model

## Abstract

Recent development has enabled synergistic drugs in treating a wide range of cancers. Being highly context-dependent, however, identification of successful ones often requires screening of combinational dose on different testing platforms in order to gain the best anticancer effects. To facilitate the development of effective computational models, we reviewed the latest strategy in searching optimal dose combination from three perspectives: (1) mainly experimental-based approach; (2) Computational-guided experimental approach; and (3) mainly computational-based approach. In addition to the introduction of each strategy, critical discussion of their advantages and disadvantages were also included, with a strong focus on the current applications and future improvements.

## Introduction

In current days, combinational drugs have been increasingly used clinically in treating various cancers. Comparing to the traditional single drug approach, combinational strategy is often found with enhancing therapeutic effects or delayed drug resistance, among which synergistic drugs are mostly desired (Chou, 2006). The past few years has witnessed the computational progress in analyzing and predicting synergistic components qualitatively (Han et al., 2017; Sarah, 2017; Sheng et al., 2017). However, the optimal dose of each component needs to be identified before the formula is clinically applied, as different dose combination may lead to different effects even for the same formula (Tallarida and Raffa, 2010). To avoid potential adverse or antagonistic effects, large-scale experiments have to be screened in a huge combinational space of drug concentration which are highly time consuming and laborious. Thus, developing smart methods either experimentally or theoretically are both in urgent need to facilitate the synergistic drug design.

Until the present time, the general experimental criteria to evaluate drug synergy mainly include Loewe isobologram (Chevereau and Bollenbach, 2015), CI index from Median Effect Principle (Chou, 2010), Bliss independence (BI) model (Bansal et al., 2014), Loewe Additivity (LA) model (Lee et al., 2007), and so on. Under defined criteria, substantial data has been accumulated which initiated the computational efforts to predict dose effects of drug combination. Despite of a few algorithm and statistical methods (Calzolari et al., 2008; Deharo and Ginsburg, 2011; Caglar and Pal, 2014; Weiss et al., 2015a), constructing quantitative model to predict synergistic dose remains highly challenging for combinational therapy. To promote future improvements in this area, we reviewed the latest progress in this area covering (1) mainly experimental-based approach; (2) Computational-guided experimental approach; and (3) mainly computational-based approach.

### Mainly Experimental-Based Approach

Normally the drug efficacy can be roughly tested via cell viability assay, such as MTT assay and various animal models. But experimental exploration of drug combinations under all dose ratio seems to be unrealistic. Any high throughput technology or heuristic design will significantly save the time and experimental costs by purposely choosing the potential candidate dose.

#### High Throughput Experimental Screening

In order to identify effective combinations of therapeutic compounds, Borisy et al. (2003) developed a high-throughput screening method to systematically screen of ∼120,000 pairwise combinations for antifungal effects in 2003. The systematic testing began by defining the activity of each compound as a single agent in the assay system. And then, each active compound against all other compounds was tested in dose matrices comprising six concentrations based on EC50. Finally, the possible synergistic dose ratio between the drug pairs would be detected. In this way, this paper proposed a practical application to systematic screening of compounds in disease-relevant phenotypic assays (Borisy et al., 2003). Furthermore, this method also proposed to detect the synergistic effects between constituents within the natural products (Isgut et al., 2017).

Then in 2007, a series of concentration ratios for each drug pair were tested on 10∼20 tumor cell lines via high-throughput screening technology (Mayer and Janoff, 2007). After analyzing the cytotoxicity curves for each, they found that certain dose ratios of combinational drugs can be synergistic, while other ratios of the same agents may be antagonistic (Mayer and Janoff, 2007). Interestingly, high-throughput screening has been applied to tumor organoids system in recent years (Ivanov and Grabowska, 2017; Ivanov et al., 2017; Shahi Thakuri and Tavana, 2017). For instance, colon cancer spheroids were applied for drug synergy between 25 compounds under multiple IC50s, instead of the traditional cell lines (Shahi Thakuri and Tavana, 2017). And animal model of zebrafish was also established for this purpose with the assistance of auto-image analysis technology (Todd et al., 2017).

#### Fixed Dose Method

To avoid random high-throughput screening, fix dose/ratio method may serve as a starting point to explore when prior information is totally unknown. The dose may be set according to their maximum tolerated doses (MTD) and partial MTDs (Cao and Rustum, 2000; Azrak et al., 2004, 2007; Cao et al., 2005). As early as in 2000, the synergistic effect of Irinotecan and 5-Fluorouracil was studied in the rat model of colon cancer, at the dose of MTDs, 12.5% MTDS, 50% MTDS, and 75% MTDS, respectively (Cao and Rustum, 2000). Another searched the synergistic effect of 200 pairs of antifungal drugs within a dose range between 0 to minimal inhibitory concentration (MIC) in the brewer’s yeast (Cokol et al., 2011). It worth to note that, besides dose combination, the time interval and sequential treatment, even the pharmaceutical packaging may influence the effects of drug combination (Azrak et al., 2007; Mohan et al., 2014).

Instead of fixed dose, some studies fixed dose ratios based IC50 when prior information is unknown (Hatakeyama et al., 2014; Zhang et al., 2014). Occasionally, dose ratio may also start from 1:1 to explore the synergistic spectrum for different drugs in different cancer types (Liu et al., 2011).

### Computational-Guided Experimental Approach

To avoid exhaustive searching in dose combinational space, computer algorithm was often adopted as a feedback control to suggest next round of experiments design based on preliminary experimental results. Current algorithms for this purpose mainly refers to feedback system control scheme (FSC), which help to converge fast in a huge searching space of multiple drugs with multiple doses. This scheme has been applied to identify the best dose combinations of multiple drugs in various cancer (Liu et al., 2015), and viral infection (Wong et al., 2008).

The procedure of FSC (Tsutsui et al., 2011; Liu et al., 2015; Weiss et al., 2015b) usually includes: (1) Input a number of drugs (usually 5 to 10) with several doses (e.g., 0, IC25, IC50, IC75) for a specific disease; (2) Combine all drugs and their doses to form a large searching space; (3) Random select partial combinations from above space and test experimentally; (4) Update the drug doses by differential evolution algorithm (DE); (5) Repeat (3) and compare latest experimental results to the previous ones; and (6) Choose better experimental results for the next iteration.

**FIGURE 1:**
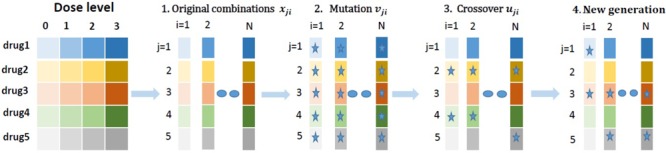
A schematic illustration of differential evolution (DE) algorithm.

Here the detailed heuristic DE algorithm (Tsutsui et al., 2011) is illustrated in **Figure 1**: (1) Choose a drug-dose combination according to a random algorithm: x_ji_; (2) Examine the effect through experiments: E(x_ji_); (3) Mutate the current selected drug dose to v_ji_; (4) Crossover the current and mutated drug-dose combination (x_ji_×v_ji_) to obtain a new drug-dose combination u_ji_; (5) Examine the effect of the new drug-dose combination: E(u_ji_); (6) Compare E(u_ji_) with E(x_ji_). The new drug-dose combination is u_ji_, if E(u_ji_) > E(x_ji_) and will go into the next iteration cycle.

It can be seen that the features of FSC as several advantages (Nowak-Sliwinska et al., 2016). Firstly, it is phenotypically driven, simpler than genotype-driven methods, and does not require any mechanism information. Secondly it can achieve a fast convergence by using DE algorithm. Despite of that, the experimental testing is still substantial because all input drugs are considered equally in the combination. Thus the improved version of FSC incorporates a regression model to identify those potential synergistic drugs out of the input list before searching optimized dose (Wang et al., 2015; Weiss et al., 2015a).

Recently, FSC was used to screen Nano-diamond modified drugs out of 57 dose combinations and therapeutic dose window was proposed which could optimally inhibit cancer cell lines and protect the normal cell lines (Wang et al., 2015). More application of FSC could be found in prostate cancer and hepatocellular carcinomas (Mohd Abdul Rashid et al., 2015; Jia et al., 2017).

### Mainly Computational-Based Approach

Apart from the above approaches, a few mathematical models have been constructed which have been collected as below.

#### Stochastic searching model

**FIGURE 2:**
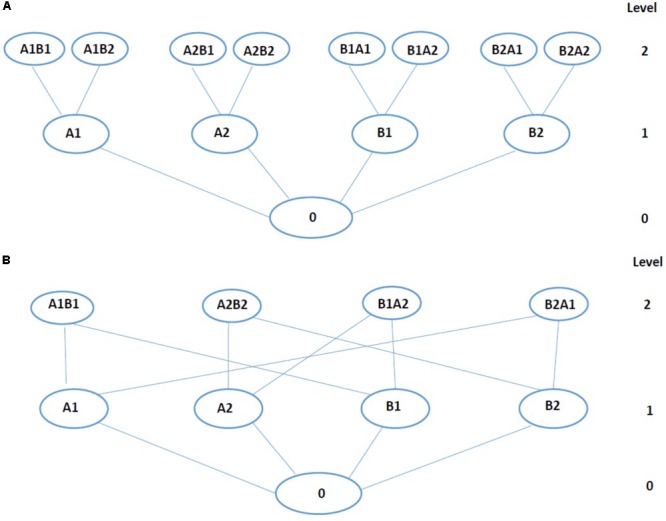
Tree representation of the data. **(A)** The tree with sequential structure. **(B)** The tree with trellis-like structure (alternative version of the **A**). Each circle stands for single drug-dose or combination. Letter and number indicates drug and dose, respectively. For a tree, level 0 is the control (no drug), level 1 is composed of individual drug treatment with two doses, and level 2 is composed of drug-dose combinations. The level depends on the size of the combination.

To minimize searching space for optimal dose combination, a few stochastic search algorithms with heuristic ideas have been reported recently (Calzolari et al., 2008; Caglar and Pal, 2014). **Figure 2A** shows an example of stochastic search algorithms, with ideas similar to that of the stack sequential algorithm (Jeline, 1969). An alternative version of the **Figure 2A** tree (**Figure 2B**), eliminates nodes representing redundant drug-dose combinations. Stochastic search algorithms works as this: under search tree structure, the biological score was evaluated at the first level of tree and best single drug Cbest was extracted (Calzolari et al., 2008). Then, the biological scores of Cbest combined with all other drugs were measured and compared with Cbest’s to decide the movements of upward or downward. The current best combination was chosen for further searching of sub-nodes to get the global optimal combinations. In this way, only one-third of the tests were actually scanned in the Drosophila model of 4 drugs (Calzolari et al., 2008).

Meanwhile, a diversified stochastic search algorithm has been recently proposed to find optimum drug concentrations efficiently without prior normalization of the searching space (Caglar and Pal, 2014). This stochastic algorithm was composed of the initial parallel part and the iteration part. The former was used to generate a rudimentary knowledge of the searching space, while the later was mainly used to search the space repeatedly to update knowledge of new hills that the previous iterations could not locate. After relatively smaller number of iterative steps, the optimized dose combination could be detected for anti-bacteria and anti-cancer effects (Caglar and Pal, 2014).

#### Statistical model

In addition to stochastic searching, statistical models were also applied to screen the optimal drug-dose combination based on cellular responses (Deharo and Ginsburg, 2011; Weiss et al., 2015a). The logistic regression model showed in equation (1) (Deharo and Ginsburg, 2011) was proposed to predict the EC50s of the drug alone and in combination. And the synergistic effects of six different ergosterol together with the pyrethroid in five selected dose ratios were detected (Deharo and Ginsburg, 2011).

(1) $$\begin{array}{c} f(x,(b,c,d,e))=c+\frac{d-c}{1+{\left(x/e\right)}^{e}} \end{array}$$

*f*(x): drug effects; *x*: dose of drug; *b*: a measure of the steepness of the curve for the dose equal to the ED50 value; *c, d*: denote the lower and upper asymptotes of the s-shaped curve; *e*: corresponds to ED50 value.

Different from the logistic regression model, the second-order linear regression model screened out the optimal drug-dose combination by firstly refine drugs which might produce synergistic effect (Chen et al., 2010; Xu et al., 2014; Weiss et al., 2015a; Silva et al., 2016). This model mainly contained the following steps: (1) Establish a stepwise linear regression model describing the relationship between drug doses and effects; (2) Select the drugs most likely to produce synergistic effects according to the model coefficients; (3) Continue to do regression analysis of the drugs selected in (2); (4) Detect final optimal drug combination and dose ratio. Through several cycles, an optimal drug combination toward viability inhibition of renal carcinoma cells from initial 10-drug pool with 4 doses each was detected (Weiss et al., 2015a).

The second-order linear regression model (Weiss et al., 2015a) is showed in equation (2)

(2) $$\begin{array}{c} y\;=\;\beta_{0}+\sum_{i=1}^{k}\beta_{i}x_{i}+\sum_{i=1}^{k}\beta_{\mathrm{ii}}x_{i}^{2}+\sum_{i=1}^{k}\sum_{j=i+1}^{k}\beta_{\mathrm{ij}}x_{i}x_{j}+\varepsilon \end{array}$$

*y*: the response variable (i.e., cell viability as percent of control); β_i_, β_ii_, β_ij_: represent the intercept and the coefficients of linear, quadratic, and bilinear terms, respectively; _x_i_, x_j__ : independent variables (i.e., drug combination at designed doses); 𝜀: an error term.

#### Multi-Scale Agent-Based Model

**FIGURE 3:**
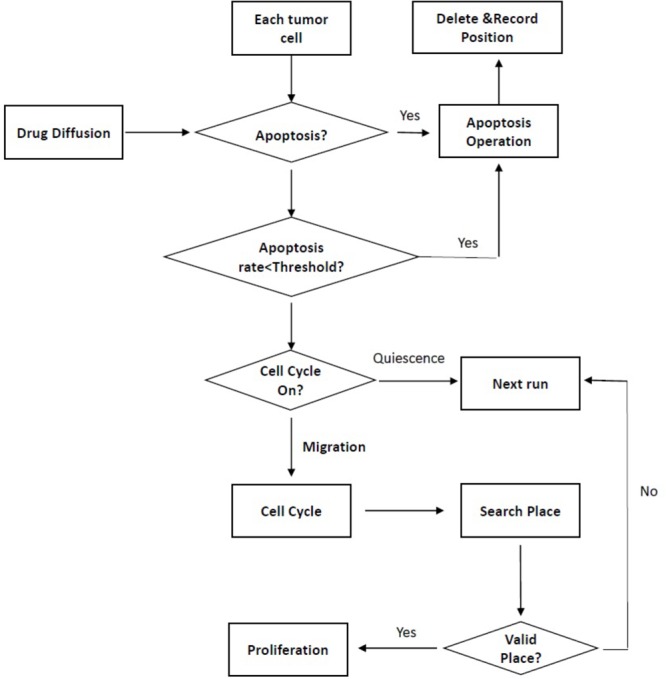
The cellular phenotype switching at the intracellular scale. Apoptosis: Under the drug diffusion, the cell will initiate apoptosis if the simulated apoptosis probability is less than the set threshold. Proliferation: The proliferation will initiate if the cell cycle is on and empty location exist to divide in the mitotic M phase. Migration: A proliferating cell will migrate in the first three phases of cell cycle (G0/G1, S, and G2) under appropriate location. Quiescence: There are two possibilities of quiescence: the cell cannot go through the cell cycle, or the cell cannot find a valid place to divide.

In recently years, the multi-scale agent-based model has been established to evaluate synergistic dose ratios by controlling the fate of cells under different drug combinations (Wang et al., 2013; Qiao et al., 2015). The model simulated the growth process of tumor cells including apoptosis, proliferation, migration, etc. based on some specialized biological regulations to screen the optimal dose combinations with maximal lethality in different dose combinations. Furthermore, the model could not only describe multicellular interaction system and microenvironment in cancer, but also detect synergistic dose with limited experimental data. Usually, the model was established according to discrete dose combination effects to simulate continuous effects under wide range of dose combinations. And the fate of cells was usually described from the intracellular, intercellular, and tissue scales to illustrate the ‘phenotypic’ switches showed in **Figure 3** (Qiao et al., 2015), cell–cell and cell–microenvironment interaction, respectively (Wang et al., 2013; Qiao et al., 2015).

In 2015, this model was firstly used to choose optimal combinations restoring the balance between osteoclast cells and osteoblast cells as well as killed cancer cells in multiple myeloma Cancer (Qiao et al., 2015). According to the pathogenesis, the behaviors of myeloma cells and two normal cells under the action of multiple cytokines and drug combinations were simulated. Ultimately, the optimal dose ratio of the combination was screened out according to the simulation result.

Besides, artificial intelligence (AI) has had an impact in drug synergy area. Recently, Preuer et al. (2017) developed a novel deep learning method, termed DeepSynergy, to model drug synergy qualitatively using chemical and genomic information, which is based on Neural Networks. This mechanism-free and data-driven method outperformed those previously methods of deep learning within the space of 38 drugs on 39 cell lines. But DeepSynergy didn’t make comparison with the other models previously reported, such as RACS (Sun et al., 2015) and other methods in DREAM Challenge (Bansal et al., 2014). RACS, which is semi-supervised, mechanism-guided, and context-dependent combining both genomic and network characteristics, showed a probability concordance of 0.78 compared with 0.61 obtained with the best algorithm reported in DREAM Challenge within the space of 14 compounds on the cell line OCI-Ly3. Furthermore, more computational approaches in qualitatively identifying synergistic drug combinations are summarized by Sheng et al. (2017). Yet AI methods have not been seen in quantitatively screening synergistic dose combinations, which worth further exploration.

## Perspective

We have summarized the latest development in the area of synergistic dose combinations for Anticancer Drugs. Above accumulated work has paved the way to comprehensive predictive model of optimal dose combination. It should be aware of that, the current searching methods are still limited to local optimization, while more experimental results are needed to validate the computational models. Although challenging, considering below factors may contribute to more effective algorithms. For instance, cancer heterogeneity should be seriously considered in order to achieve better results. Meanwhile, considering the drug response of multiple cells/tissues may minimize the potential side effects of combined drugs to normal tissues. This is particularly important when the drugs are administrated with different time and different order. Coupled with the future development of AI and hardware development, more concrete models are expected to potentially assist the clinical decision of combinational drug dosage to cancer patients.

## Author Contributions

ZY collected the main papers and wrote the manuscript. ZD and WZ collected the related studies. ZC supervised the whole project and modified the manuscript. All authors read the approved the final manuscript.

## Conflict of Interest Statement

The authors declare that the research was conducted in the absence of any commercial or financial relationships that could be construed as a potential conflict of interest.
